# High-Dimensional Analysis of Finger Motion and Screening of Cervical Myelopathy With a Noncontact Sensor: Diagnostic Case-Control Study

**DOI:** 10.2196/41327

**Published:** 2022-10-03

**Authors:** Takafumi Koyama, Ryota Matsui, Akiko Yamamoto, Eriku Yamada, Mio Norose, Takuya Ibara, Hidetoshi Kaburagi, Akimoto Nimura, Yuta Sugiura, Hideo Saito, Atsushi Okawa, Koji Fujita

**Affiliations:** 1 Department of Orthopaedic and Spinal Surgery Graduate School of Medical and Dental Sciences Tokyo Medical and Dental University Tokyo Japan; 2 School of Science for Open and Environmental Systems Graduate School of Science and Technology Keio University Kanagawa Japan; 3 Department of Functional Joint Anatomy Graduate School of Medical and Dental Sciences Tokyo Medical and Dental University Tokyo Japan

**Keywords:** cervical myelopathy, myelopathy, spinal cord disease, spinal cord disorder, nervous system disorder, nervous system disease, clumsiness, screening, 10-second hand grip and release test, machine learning, carpal tunnel syndrome, Leap Motion, clinical informatics, system validation, screening system, sensor, model, diagnosis, diagnostic, high-dimensional analysis, motion sensor, motion detection, high-dimensional data analysis, high-dimensional statistics

## Abstract

**Background:**

Cervical myelopathy (CM) causes several symptoms such as clumsiness of the hands and often requires surgery. Screening and early diagnosis of CM are important because some patients are unaware of their early symptoms and consult a surgeon only after their condition has become severe. The 10-second hand grip and release test is commonly used to check for the presence of CM. The test is simple but would be more useful for screening if it could objectively evaluate the changes in movement specific to CM. A previous study analyzed finger movements in the 10-second hand grip and release test using the Leap Motion, a noncontact sensor, and a system was developed that can diagnose CM with high sensitivity and specificity using machine learning. However, the previous study had limitations in that the system recorded few parameters and did not differentiate CM from other hand disorders.

**Objective:**

This study aims to develop a system that can diagnose CM with higher sensitivity and specificity, and distinguish CM from carpal tunnel syndrome (CTS), a common hand disorder. We then validated the system with a modified Leap Motion that can record the joints of each finger.

**Methods:**

In total, 31, 27, and 29 participants were recruited into the CM, CTS, and control groups, respectively. We developed a system using Leap Motion that recorded 229 parameters of finger movements while participants gripped and released their fingers as rapidly as possible. A support vector machine was used for machine learning to develop the binary classification model and calculated the sensitivity, specificity, and area under the curve (AUC). We developed two models, one to diagnose CM among the CM and control groups (CM/control model), and the other to diagnose CM among the CM and non-CM groups (CM/non-CM model).

**Results:**

The CM/control model indexes were as follows: sensitivity 74.2%, specificity 89.7%, and AUC 0.82. The CM/non-CM model indexes were as follows: sensitivity 71%, specificity 72.87%, and AUC 0.74.

**Conclusions:**

We developed a screening system capable of diagnosing CM with higher sensitivity and specificity. This system can differentiate patients with CM from patients with CTS as well as healthy patients and has the potential to screen for CM in a variety of patients.

## Introduction

Cervical myelopathy (CM) occurs in patients with cervical spondylotic myelopathy, ossification of the posterior longitudinal ligament, or cervical disk herniation [1-3]. CM causes symptoms such as clumsiness of the hands, numbness of the extremities and trunk, and gait disturbance, and often requires surgery. The longer the duration and the more severe the disease, the worse the postoperative outcome [4-6]. However, some patients with CM are unaware of their early symptoms and consult a spine surgeon only after their condition has become severe [7]. Therefore, screening and early diagnosis of CM are important for symptom monitoring and to determine the optimum time for surgery [8].

Clumsiness of hands is a characteristic and important symptom of CM and is referred to as myelopathy hand [9]. The 10-second hand grip and release (10-s) test is commonly used to check for the presence of myelopathy hand [9,10]. In the 10-s test, patients repeatedly grip and release their hand as fast as possible for 10 seconds; if the number of repetitions is less than 20, a myelopathy hand is suspected. The 10-s test is simple but would be more useful for screening if it could objectively evaluate not only the number of repetitions but also the changes in movement specific to myelopathy hand.

Nowadays, the latest commercial sensors and devices using virtual reality have been developed and are being used in the medical field [11]. Some studies have reported using smartphones and stylus pens to analyze hand movements and diagnose diseases [12-14]. In the field of cervical spine, there have been reports of diagnosis, surgery, and rehabilitation using virtual reality [15-17]. Several studies have also been conducted to analyze the movement of the myelopathy hand using sensors [18-22]. Most of these studies used wearable sensors such as motion capture systems, strain sensors, gyro sensors, and bend sensors, which are complicated.

For a simpler test, we analyzed hand and finger movements in the 10-s test using Leap Motion (Leap Motion) in a previous study [23]. Leap Motion is a noncontact sensor consisting of infrared cameras and LEDs, and captures hand and finger movements in real time [24,25]. Furthermore, we applied a machine learning algorithm to the obtained data to create a binary classification model to classify CM with 84% sensitivity, 60.7% specificity, and 0.85 area under the curve (AUC). However, because of the limitations of the system, only fingertip movements, not all joint movements, were recorded. Moreover, because only patients with CM and healthy participants were compared, it was not clear whether our model could differentiate CM from other hand disorders such as carpal tunnel syndrome (CTS).

To solve these problems, we improved the system so that the joints of each finger can also be recorded by Leap Motion and aimed to develop a system capable of diagnosing CM with higher sensitivity and specificity. Furthermore, we included patients with CTS, a common hand disorder, to verify if it is possible to distinguish CM from CTS.

## Methods

### Ethics Approval

This study was approved by the Institutional Review Board of Tokyo Medical and Dental University (M2019-047). Written informed consent was provided by all participants.

### Recruitment

We included preoperative patients with CM (CM group), preoperative patients with CTS (CTS group), and volunteers (control group) between February 2020 and July 2021. Experienced spine surgeons diagnosed CM based on symptoms, physical and neurological findings, and magnetic resonance imaging (MRI) or computed tomography myelogram. Experienced hand surgeons diagnosed CTS based on symptoms, physical findings such as the Tinel sign and Phalen test, and nerve conduction studies (NCSs) measured by Neuropack X1 (Nihon Kohden). Volunteers were recruited from patients who had undergone total hip arthroplasty.

In all groups, participants with a history of other upper extremity disease, injury, or surgery; those with neurological diseases such as stroke, brain tumor, and traumatic brain injury; those with inflammatory diseases such as rheumatoid arthritis; those with dementia or psychiatric disease; and those who refused to participate were excluded. Moreover, spine surgeons also examined participants in the CTS and control groups, and excluded those with symptoms or physical findings suggestive of CM from the CTS and control groups. Similarly, hand surgeons examined participants in the CM and control groups, and excluded those with symptoms or physical findings suggestive of CTS from the CM and control groups.

In the CM group, primary diseases causing CM were recorded. The maximally compressed levels of the spinal cord were also recorded from the sagittal and axial images of the preoperative T2-weighted MRI. In the CTS group, Bland classifications were recorded as severity based on NCSs [26]. Finally, the CTS and control groups were combined to create a non-CM group.

### Measurements With Leap Motion

**Figure 1:**
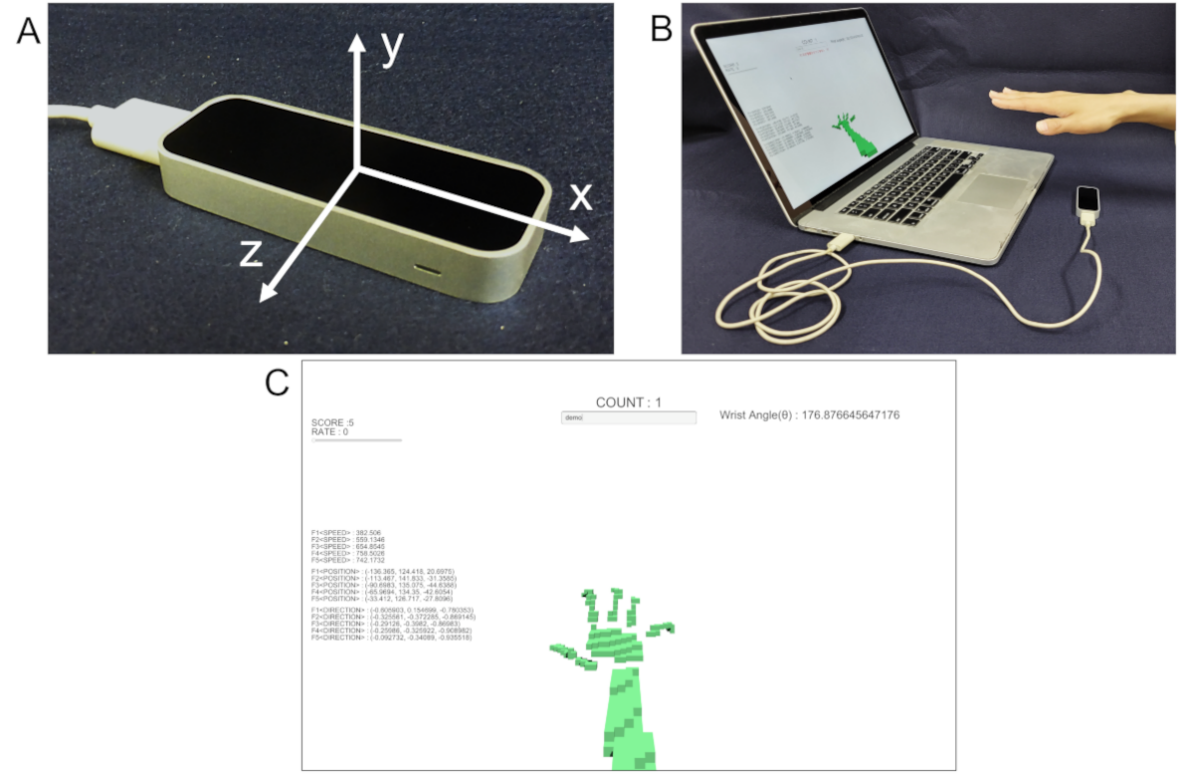
Images of the measurement with Leap Motion. Leap Motion and the three axes measured by Leap Motion (A). Participants placed their hand above Leap Motion, connected to a laptop computer via USB (B). During the measurement, a 3D hand model was displayed in real time on the screen of the laptop computer (C).

Before the measurement, the procedure and a short demonstration were provided to the participants. The protocol of the measurement with Leap Motion was based on a previous study and was performed as follows: participants sat in front of Leap Motion placed in front of a laptop computer and connected by USB, extended the elbow on the side to be measured, placed the hand 10 cm above Leap Motion in a pronated position, and gripped and released the fingers as rapidly and as fully as possible 20 times after seeing the sign to start the examination (Figure 1) [23]. During the measurement, we confirmed that the system could correctly capture participant hand movements by watching the 3D hand model displayed on the screen in real time. All participants completed both hand measurements twice. A total of 229 parameters, listed in Table 1, were measured as waveform data (60 frames per second).

**Table 1:** Parameters measured by Leap Motion.

Parameters	Values, n	Total (N=229), n
Extended fingers (n)	1	1
Position of palm	3 dimensions^a^	3
Direction of palm	3 dimensions	3
Angle of wrist extension	1	1
Position of wrist	3 dimensions	3
Direction of forearm	3 dimensions	3
Speed of fingertip	5 fingers	5
Position of fingertip	5 fingers × 3 dimensions	15
Direction of fingertip	5 fingers × 3 dimensions	15
Position of distal end of bone	5 fingers × 4 bones^b^ × 3 dimensions	60
Position of center of bone	5 fingers × 4 bones × 3 dimensions	60
Direction of bone	5 fingers × 4 bones × 3 dimensions	60

^a^Dimensions consist of x, y, and z coordinates.

^b^Bones consist of distal phalanx, middle phalanx, proximal phalanx, and metacarpus. For convenience, bones of the thumb were assumed to consist of distal phalanx, proximal phalanx, metacarpus, and carpal bones.

### Statistical Analysis

#### Characteristics of Participants

The characteristics of participants were assessed using Student *t* test for age, chi-square test for sex and measured side of the hand, and Fisher exact test for hand dominance. A *P* value <.05 was considered statistically significant.

#### Binary Classification Model

We aimed to create two models, one to diagnose CM among the CM and control groups (CM/control model), and the other to diagnose CM among the CM and non-CM groups (CM/non-CM model).

Preprocessing of the data was performed prior to the application of machine learning. First, each waveform data was divided into 15 segments of 64 frames each while allowing for overlap because each participant took different frames to perform 20 grips and releases. These segments (64 frames) were linearly detrended and multiplied by the Hanning window function [27]. The processed segments were converted to frequency domain data using fast Fourier transform. The subwaveforms (64 frames) were converted into frequency domain data, selecting only the lower 16 frequencies. Finally, a 54,960-dimensional data set (229 parameters × 16 frequency domain data × 15 segments) was obtained for each trial. Data from two trials on each hand were combined and used to create the CM/control model. Alternatively, since CTS can occur on only one hand, data from only two trials on one hand (either the right or left) were combined and used to create the CM/non-CM model.

A support vector machine (SVM) was used to create the binary classification models [28]. SVM is one of the common machine learning algorithms used for classification and has performed well in previous studies. After the learning phase, the SVM shows a predicted label of CM with a probability score. We set a threshold and created a binary classification model to classify whether a data set was CM or not. Data from the CM and control groups were used for the CM/control model, and data from all groups were used for the CM/non-CM model. In the validation phase, 10-fold cross-validation was performed [29]. We generated a receiver operating characteristic (ROC) curve by adjusting the threshold and calculating the AUC. The point on the ROC curve closest to the upper-left corner of the graph was set as the optimal cutoff value.

Furthermore, to investigate which parts of the hand contribute to the diagnosis of CM, we also generated modified CM/control models using data from only one of the 20 bones and then similarly calculated the AUC.

## Results

### Comparison of Characteristics of Participants

In total, 31 participants (62 hands), 27 participants (38 hands), and 29 participants (58 hands) were recruited to the CM, CTS, and control groups, respectively. Patient demographics and characteristics are summarized in Table 2. There was no significant difference between the groups in terms of age, sex, or hand dominance.

**Table 2:** Characteristics of participants in the CM, CTS, and control groups.

Characteristic			Non-CM^a^					CM			*P* value		
			Control		CTS^b^					CM/control			CM/non-CM
Participants, n			29		25		31			N/A^c^			N/A
Age (years), mean (SD)			63.6 (52.1-75.0)		62.0 (49.2-74.7)		67.0 (57.0-77.0)			.23			.11
Sex (male), n			12		5		16			.59			.11
Hand dominance (right), n			29		25		30			>.99			.36
Hands, n			58		34		62			N/A			N/A
Side (right), n			29		20		31			>.99			.83
**Bland classification, n**			N/A				N/A			N/A			N/A
	Grade 1			3									
	Grade 2			0									
	Grade 3			17									
	Grade 4			0									
	Grade 5			14									
	Grade 6			4									
**Primary disease, n**			N/A		N/A					N/A			N/A
	CSM^d^					13							
	OPLL^e^					16							
	CDH^f^					2							
**Maximally compressed level, n**			N/A		N/A					N/A			N/A
	C1/2					1							
	C2/3					0							
	C3/4					12							
	C4/5					8							
	C5/6					9							
	C6/7					1							

^a^CM: cervical myelopathy.

^b^CTS: carpal tunnel syndrome.

^c^N/A: not applicable.

^d^CSM: cervical spondylotic myelopathy.

^e^OPLL: ossification of the posterior longitudinal ligament.

^f^CDH: cervical disk herniation.

### Binary Classification Model

**Figure 2:**
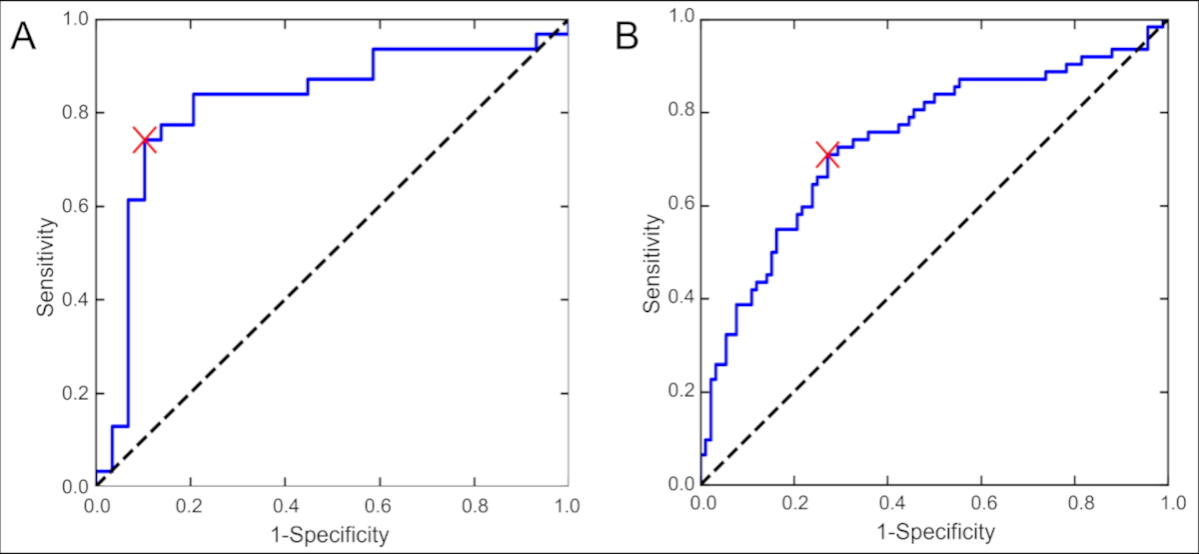
Receiver operating characteristic (ROC) curve of the cervical myelopathy (CM)/control model (A) and CM/non-CM model (B). The area under the ROC curve was 0.82 and 0.74 in the CM/control model and CM/non-CM model, respectively. The red cross indicates the optimal cutoff value.

The indexes of the binary classification models are listed in Table 3. The ROC curve of the control and CM/non-CM model are shown in Figure 2.

The AUC of models limited to the parameters of each bone are listed in Table 4. The AUC of the model using the parameters of the proximal phalanx of the thumb was the highest (0.86).

**Table 3:** Index of binary classification models.

		Sensitivity (%)	Specificity (%)	AUC^a^
CM^b^/control model		74.2	89.7	0.82
**CM/non-CM model**				
	Total	71.0	72.8	0.74
	Right hand	71.0	75.5	0.77
	Left hand	74.2	79.1	0.76

^a^AUC: area under the curve.

^b^CM: cervical myelopathy.

**Table 4:** Area under the curve of models limited to the parameters of each bone

	Thumb^a^	Index finger	Middle finger	Ring finger	Little finger
Distal phalanx	0.83	0.82	0.80	0.78	0.78
Middle phalanx	0.86	0.83	0.81	0.80	0.79
Proximal phalanx	0.84	0.82	0.84	0.83	0.83
Metacarpus	0.82	0.82	0.83	0.83	0.82

^a^Only in the thumb, middle phalanx means proximal phalanx, proximal phalanx means metacarpus, and metacarpus means carpal bones.

## Discussion

### Principal Results

We developed a classification model with high sensitivity and specificity to diagnose CM. However, despite increasing the parameters, major improvements in diagnostic performance of the CM/control model were not obtained in this study (74.2% sensitivity, 89.7% specificity, and 0.82 AUC) compared to the previous study (84% sensitivity, 60.7% specificity, and 0.85 AUC) [23]. Increasing only the number of parameters will result in improved diagnostic performance; therefore, it is necessary to increase the number of samples. Nevertheless, the classification model in this study is still effective as a screening method since it has a sufficiently high diagnostic performance when compared to classic tests. For example, the 10-s test showed 61%-74% sensitivity, 52%-66% specificity, and 0.71-0.77 AUC [10,30,31]; the finger escape sign showed 48%-55% sensitivity [30,31]; the deep tendon reflex change showed 15%-56% sensitivity and 96%-98% specificity [31-33]. In another previous study, the analysis and diagnoses of myelopathy hand was performed by wearing a glove with a sensor, with 87% sensitivity, 86% specificity, and 0.93 AUC [21]. Although the result of this study is inferior to the previous study, our method is superior in that it is easier to test many patients with the noncontact sensor, making it suitable for screening.

In the models limited to the parameters of each bone, the AUC of the model using the parameters of the proximal phalanx of the thumb was the highest. In addition, overall, the models using the parameters of bones of the thumb tended to have higher AUCs. This result is contrary to the finger escape sign, which indicates that the ulnar finger is more likely to be affected in CM [9]. The cause of this discrepancy may be due to the position of the sensor in this method. Because Leap Motion captures hand movement from the palmar side, the bones of the fingers other than the thumb are temporarily hidden by other bones during the grip and release movements, and occasionally not accurately captured. Alternatively, thumb movement is always tracked by Leap Motion. Moreover, another study reported that patients with CM exhibit specific changes in pinching movements with the thumb and index finger [20]. This result means that, in patients with CM, not only ulnar but also radial finger movements are significantly altered. These factors would contribute to the higher AUCs of the model using the parameters of the proximal phalanx of the thumb.

In this study, we attempted to differentiate the CM group from not only the control group, as in the previous study [23], but also the CTS (non-CM) group, and we achieved high diagnostic performance. The peak onset of CM is between the years of 40 and 60 years [1,3], but other hand disorders are also prevalent during that time. Because CTS is a common hand disorder, with a predilection for people 40 years or older [34,35], we included these patients in our study. Our system can distinguish myelopathy hand from motor disorders of the thumb that can occur in CTS [36]. While further trials are required to differentiate CM from other hand disorders, this result suggests the possibility of accurately screening for CM among a variety of hand disorders.

Several studies have also been conducted to analyze the movement of the myelopathy hand using sensors, but Leap Motion has the major advantage of simplicity. For example, motion captures can provide a detailed motion analysis, but the installation of the sensors requires skill and time of the examiners, and it is impossible to test a large number of patients in a short period of time. Alternatively, Leap Motion can be used for our test simply by connecting it to a computer if the program can be shared. Furthermore, the test can be performed by a single patient with only a simple test procedure guide. Leap Motion is also a less expensive commercial sensor, which is an advantage in that it is readily available. These advantages of Leap Motion are useful for screening large numbers of patients in a short period of time.

### Limitations

This study had some limitations. First, it is possible that there were participants with potential CM in the CTS and control groups because participants in these groups did not undergo an MRI. Similarly, it is possible that there were participants with potential CTS in the CM and control groups because participants in these groups did not undergo an NCS.

Second, we did not compare subgroups by anatomical level of myelopathy and by severity of CM and CTS. There may be variation among subgroups within the same group. Third, only internal validation by 10-fold cross validation was performed and external validation was not. In future work, we will collect more samples to solve these problems.

### Conclusions

We developed a screening system capable of diagnosing CM with higher sensitivity and specificity by high-dimensional analysis of finger motion and machine learning. This system can differentiate patients with CM from patients with CTS as well as healthy patients and has the potential to screen for CM in a variety of patients.

## Abbreviations

10-s: 10-second hand grip and release
AUC: area under the curve
CM: cervical myelopathy
CTS: carpal tunnel syndrome
MRI: magnetic resonance imaging
NCS: nerve conduction study
ROC: receiver operating characteristic
SVM: support vector machine
